# Key sunitinib‐related biomarkers for renal cell carcinoma

**DOI:** 10.1002/cam4.4206

**Published:** 2021-08-17

**Authors:** Yun Peng, Shiqiang Dong, Yuxuan Song, Dingkun Hou, Lili Wang, Bowen Li, Haitao Wang

**Affiliations:** ^1^ Tianjin Institute of Urology The 2nd Hospital of Tianjin Medical University Tianjin China; ^2^ Department of Urology Tianjin Medical University General Hospital Tianjin China; ^3^ Department of Oncology The 2nd Hospital of Tianjin Medical University Tianjin China

**Keywords:** CEACAM4, CRYBB1, HAMP, renal cell carcinoma, sunitinib

## Abstract

**Background:**

Renal cell carcinoma (RCC) contributed to 403,262 new cases worldwide in 2018, which constitutes 2.2% of global cancer, nevertheless, sunitinib, one of the major targeted therapeutic agent for RCC, often developed invalid due to resistance. Emerging evidences suggested sunitinib can impact tumor environment which has been proven to be a vital factor for tumor progression.

**Methods:**

In the present study, we used ssGSEA to extract the immune infiltrating abundance of clear cell RCC (ccRCC) and normal control samples from GSE65615, TCGA, and GTEx; key immune cells were determined by Student's *t*‐test and univariable Cox analysis. Co‐expression network combined with differentially expressed analysis was then applied to derive key immune‐related genes for ccRCC, followed by the identification of hub genes using differential expression analysis. Subsequently, explorations and validations of the biological function and the immune‐related and sunitinib‐related characteristics were conducted in KEGG, TISIDB, Oncomine, ICGC, and GEO databases.

**Results:**

We refined immature dendritic cells and central memory CD4 T cells which showed associations with sunitinib and ccRCC. Following, five hub genes (CRYBB1, RIMBP3C, CEACAM4, HAMP, and LYL1) were identified for their strong relationships with sunitinib and immune infiltration in ccRCC. Further validations in external data refined CRYBB1, CEACAM4, and HAMP which play a vital role in sunitinib resistance, immune infiltrations in ccRCC, and the development and progression of ccRCC. In conclusion, our findings could shed light on the resistance of sunitinib in ccRCC and provide novel biomarkers or drug targets for ccRCC.

## Graphical abstract

We identified five hub genes (CRYBB1, RIMBP3C, CEACAM4, HAMP, and LYL1) related to immune infiltration, sunitinib in renal carcinoma. External validation refined CRYBB1, CEACAM4 and HAMP which played a vital role in the development and progression of ccRCC and implicated in sunitinib resistance and immune infiltrations in ccRCC.

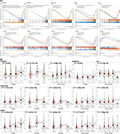

## 1 INTRODUCTION

Cancer of the kidney led to 403,262 new cases worldwide in 2018, which constituted 2.2% of global cancer.^1^ Renal cell carcinoma (RCC) has various histological subtypes with a unique molecular landscape for each sort, of which clear cell renal cell carcinoma (ccRCC) is the most prevalent subtype and contributes to 75% of all RCC cases.^2^ Sunitinib, a tyrosine kinase inhibitor (TKI), was the main targeted therapeutic agent for ccRCC in the last decade,^2^, ^3^ nevertheless, most patients develop resistance in 6–15 months.^4^ Thereby, further understanding of the action of sunitinib on ccRCC is necessary for the improvement of therapeutic for ccRCC.

Recently, tumor microenvironment (TME), including immune cells, inflammatory cells, and fibroblasts, plays a vital role in tumor growth and progression. Different sorts of carcinoma accompanied diverse TME in which communications can promote tumor development and metastasis.^5^, ^6^ Meanwhile, increasing evidences suggested that TKIs can remodel the vascular network or the immune component in TME.^7^ Sunitinib can inhibit the development of tumor disordered vessels and induce vascular normalization. In addition, sunitinib was revealed to influence the infiltration of regulatory T cells, M2 macrophages, and CD4^+^ or CD8^+^ T cells.^7^, ^8^, ^9^, ^10^ Nevertheless, limited comprehensive analysis has been conducted to reveal the impact of sunitinib on TME in ccRCC, the present study aimed to explore specific‐TME of ccRCC and identify biomarkers for the synergy with sunitinib.

In this study, we used single‐sample Gene Set Enrichment Analysis (ssGSEA)^11^, ^12^ to enumerate immune infiltration levels of 28 immune cell sorts in ccRCC samples and normal control samples from GSE65615,^13^ The Cancer Genome Atlas (TCGA), and Genotype‐Tissue Expression (GTEx) databases. Student's *t*‐test and univariable Cox analysis were applied to derive key immune cells for ccRCC. Subsequently, Co‐expression networks were developed in ccRCC samples, followed by the identification of immune‐related genes most associated with sunitinib and ccRCC. Hub genes were further identified by differential expression analysis comparing sunitinib‐treated ccRCC with untreated ccRCC. We then explored the biological functions of hub genes and the associations with tumor stages. Finally, we validated the associations with sunitinib and immune in Kyoto Encyclopedia of Genes and Genomes (KEGG),^14^ GEO, and tumor–immune system interactions (TISIDB)^15^ databases, respectively. Our findings could shed light on the resistance of sunitinib in ccRCC and provide novel biomarkers or drug targets for ccRCC.

## 2 MATERIALS & METHODS

### 2.1 Data source and Data preprocessing

Gene expression data of 122 ccRCC samples, which were sunitinib untreated (*n* = 47) or treated (*n* = 75) before cytoreductive nephrectomy, were derived from GSE65615 ^13^ in GEO database. The RNA‐seq raw counts and FPKM values data, along with detailed clinical information, including 539 ccRCC and 72 normal control samples, were downloaded from TCGA database^16^; after filtering 8 replicated samples and 8 samples without corresponding clinical data, a total of 523 ccRCC and 72 normal control samples were applied to downstream analysis. Furthermore, the raw RNA‐seq counts and TPM values of 85 normal control kidney samples were obtained from GTEx database,^17^ we also confirmed no duplicated samples in GTEx in subsequent analysis. GSE29609 ^18^ and GSE73731 ^19^ datasets and RECA‐EU^20^ dataset were also downloaded from GEO database and ICGC^20^ database, respectively, for the validation of our findings. The approval from the ethics committee and informed consent were waived as the data in this study came from the GEO, TCGA, GTEx, and ICGC databases. We used the R program (version: 4.0.5)^21^ for the analysis of most of our study.

### 2.2 Exploring the relationships between sunitinib and immune infiltration

MSigDB^22^ immunologic signature gene sets (C7) were searched to investigate the association between sunitinib and immune using Gene Set Enrichment Analysis (GSEA).^23^ Genes were ranked decreasingly according to the log2FoldChange, which compared sunitinib‐treated and sunitinib‐untreated ccRCC by limma,^24^ clusterProfiler^25^ was then utilized to implement GSEA algorithm, and adjusted *p* < 0.25, minimal gene sets 15, and maximal gene sets 500 were regarded as the cutoff which is recommended by GSEA for exploratory analysis.^26^

### 2.3 Immune cell infiltrating abundance

The infiltrating levels of 28 immune cell sorts were enumerated using ssGSEA^11^, ^12^ as many studies^27^, ^28^, ^29^, ^30^, ^31^ did, an enrichment score from ssGSEA analysis was used to represent the infiltration abundance of immune cell, the enrichment score was scaled to unity distribution, so the minimal of the score is zero and the maximal is one. The gene expression data were first rank normalized and then employed into ssGSEA analysis. The immune cell infiltration levels of ccRCC and normal control samples were all estimated.

### 2.4 Significant immune cell sorts related to sunitinib in ccRCC

The different infiltration abundances were tested by student's *t*‐test, survival implications of each immune cell sort were analyzed by univariable Cox analysis with reference to overall survival (OS) and progression‐free interval (PFI),^32^ OS represents the period from the date of diagnosis until the date of death from any cause; PFI stands for the period from the date of diagnosis until the date of the first occurrence of a new tumor event; a cutoff of *p* < 0.05 represented statistical significance. Only immune cell types, which infiltrated differently in both comparisons between sunitinib‐treated ccRCC and untreated ccRCC and between ccRCC samples and normal control samples, meanwhile, they should significantly implicate in both OS and PFI of ccRCC, were then employed to downstream analysis.

### 2.5 Co‐expression network analysis and immune‐related genes

Co‐expression network analysis was utilized to identify genes most related to immune infiltration using WGCNA.^33^, ^34^ Gene significance (GS), module significance (MS), and module membership (MM) were defined by biweight midcorrelation coefficients. Genes in modules with maximal MS were regarded as immune‐related genes for downstream analysis^35^, ^36^ as many studies did.^37^, ^38^, ^39^, ^40^, ^41^ A threshold of 5 for softpower, 25 for minModuleSize, and 0.20 for mergeCutHeight, was used to explore the gene co‐expression network among the top 15,000 genes with maximal median absolute deviation (MAD) in ccRCC samples treated with sunitinib. With regard to the construction of the network in ccRCC samples from TCGA, we first identified differentially expressed genes comparing ccRCC with normal control samples from TCGA and GTEx using DESeq2,^42^ genes with a threshold of |log2FoldChange| >1 and adjusted *p* < 0.01 were considered as statistically significant. Subsequently, genes with a zero MAD were filtered, which yielded 8752 genes for the development of the co‐expression network with a parameter of softpower 7, minModuleSize 25, and mergeCutHeight 0.20.

### 2.6 Determination of hub genes

We first acquired the candidate immune‐related genes by intersecting sunitinib‐immune‐related genes with ccRCC‐immune‐related genes from the respective co‐expression network. Limma was then used to identify differential expression genes between sunitinib‐treated ccRCC and untreated ccRCC with a cutoff of adjusted *p* < 0.05, which contributed to the identification of five hub genes.

### 2.7 Exploring the biological process of hub genes

The hallmark gene sets in MSigDB^22^ were first utilized to dissect the immune, proliferation, and signaling pathway hub genes implicated in using GSEA. A heatmap implemented by ComplexHeatmap package^43^ was used to depict the results of GSEA. GSEA was also utilized to determine the biological process and functional pathways of hub genes by clusterProfiler^25^ R package. Adjusted *p* < 0.05 was regarded as the cutoff value. The expression levels of hub genes across varied tumor stages were explored. t‐test and analysis of variance were applied to test the statistical difference.

### 2.8 Exploring the importance of hub genes to ccRCC

Oncomine^44^ was first analyzed to explore the differential expression of hub genes in varied tumor types with a threshold of *p* < 0.05. Log‐rank analysis was further used to determine the association with OS, PFI, or disease‐specific survival (DSS) in TCGA, ICGC RECA‐EU, and GSE29609 datasets based on groups separated by the median expression level of each hub gene.

### 2.9 Validation of sunitinib–immune‐related characteristics of hub genes

To validate the association with sunitinib, sunitinib‐related pathways (MAPK signaling pathway, VEGF signaling pathway, and pathways in cancer)^45^ were searched in KEGG,^14^ and GSEA was used to analyze the enrichment of hub genes in these pathways with *p* < 0.05 regarded as the statistical significance. Subsequently, immune subtypes information of ccRCC and genes coding immunomodulators and chemokines were collected from Thorsson's study^46^ and Charoentong's study^27^ respectively. A total of six immune subtypes including C1 (wound healing), C2 (IFN‐gamma dominant), C3 (inflammatory), C4 (lymphocyte depleted), C5 (immunologically quiet), and C6 (TGF‐b dominant) were found. Kruskal–Wallis rank sum test was used to explore the differential expression of hub genes across different immune subtypes. Spearman correlation coefficient analysis was utilized to determine the association between hub genes and genes coding immunomodulators and chemokines. Furthermore, the relationship of hub genes with the abundance of tumor‐infiltrating lymphocytes across different tumor types in TCGA was investigated in TISIDB. We also employed GSE73731 ^19^ dataset to explore the above correlation for hub genes in ccRCC.

### 2.10 Statistical analysis

All statistical tests were based on a significant *p* < 0.05, Benjamini–Hochberg method was used to adjust the *P*‐value followed by a cutoff of adjusted *p* < 0.05 when it is involved in multiple comparisons problem, except for exploratory analysis using GSEA to reveal the relationships between sunitinib and immune where adjusted *p* < 0.25 recommended by GSEA^26^ was used as the cutoff.

## 3 RESULTS

**FIGURE 1:**
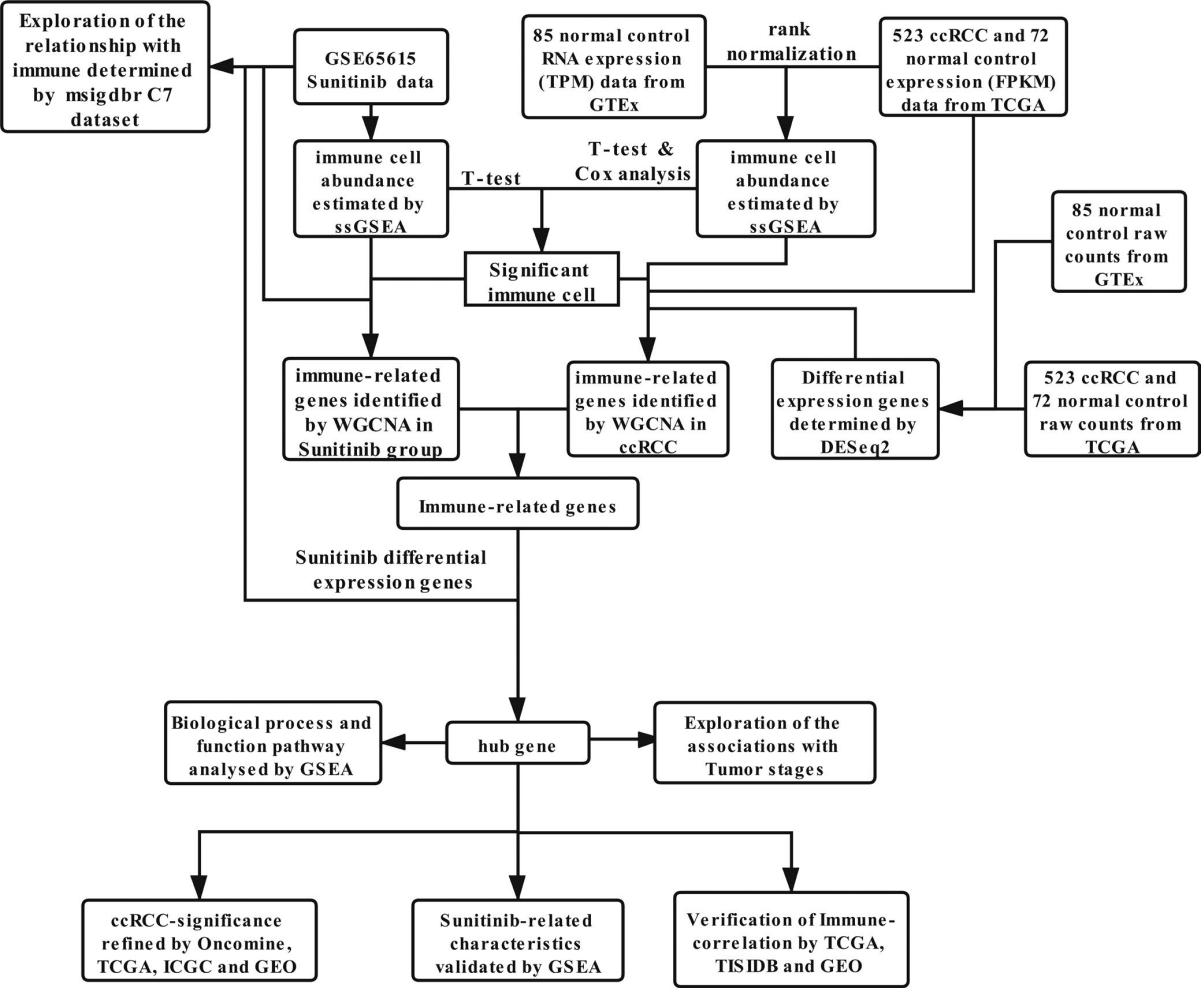
The workflow of the identification of sunitinib‐related immunotherapeutic biomarkers for renal cell carcinoma

The workflow of this study is depicted in Figure 1.

### 3.1 Key immune infiltrating cells

**FIGURE 2:**
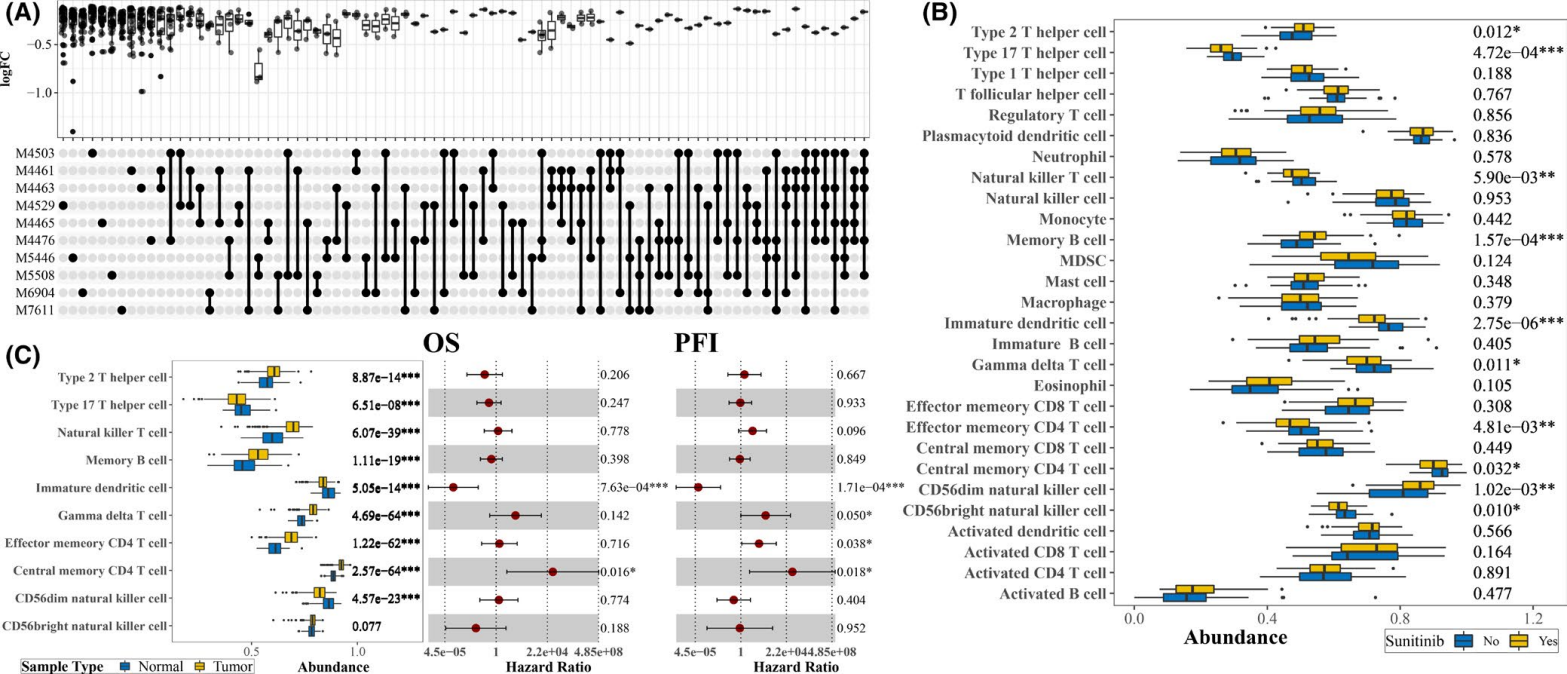
Identification of key immune cells for ccRCC. (A) The upset plot for the GSEA results in MSigDB immunologic gene set where the y‐axis represented the log2FoldChange comparing sunitinib‐treated ccRCC with untreated ccRCC. M4503 genes down regulated in comparison of naive CD4 [GeneID = 920] T cells versus unstimulated dendritic cells (DC). M4461 genes downregulated in comparison of monocytes cultured for 0 days versus those cultured for 7 days. M4463 genes downregulated in comparison of monocytes cultured for 1 day versus those cultured for 7 days. M4529 genes downregulated in comparison of naive CD4 [GeneID = 920] T cells versus stimulated CD4 [GeneID = 920] Th2 cells at 48 h. M4465 genes downregulated in comparison of neutrophils versus dendritic cells. M4476 genes downregulated in comparison of naive CD4 [GeneID = 920] CD8 T cells versus unstimulated dendritic cells. M5446 genes up regulated in comparison of mast cells versus central memory CD4 [GeneID = 920] T cells. M5508 genes upregulated in comparison of macrophages versus NK cells. M6904 genes upregulated in macrophages with IL10 [GeneID = 3586] knockout treated by LPS: 10 min versus 30 min. M7611 genes upregulated in memory CD8 T cells: 2' versus 3'. (B) The differential infiltration abundances comparing sunitinib‐treated ccRCC with untreated ccRCC were tested by student's *t*‐test, label (*) means *p* < 0.05, label (**) means *p* < 0.01, and label (***) means *p* < 0.001. (C) Left panel showed the boxplot of the immune infiltration levels of differential infiltration immune cell between ccRCC and normal control samples, student's *t*‐test was also used to test the differential infiltration between ccRCC samples and normal control samples, middle and right panels depicted the forest plot for the univariable Cox analysis for OS and PFI, respectively, label (*) means *p* < 0.05, label (**) means *p* < 0.01, and label (***) means *p* < 0.001

GSEA was first applied to reveal the relationships between sunitinib and TME. A total of 4872 immune gene sets were examined, which led to a statistically significant enrichment in 3718 gene sets, the most related 10 gene sets are depicted in an Upsetplot (Figure 2A). Following, the immune infiltration levels of 28 immune cell types were quantified by ssGSEA (Table S1 and Table S2), Student's *t*‐test showed CD56bright natural killer cell, CD56dim natural killer cell, central memory CD4 T cell, effector memory CD4 T cell, gamma delta T cell, immature dendritic cell, memory B cell, natural killer T cell, Type 17 T helper cell, and Type 2 T helper cell were infiltrating differently between sunitinib‐treated ccRCC and untreated ccRCC (Figure 2B and Table S3). Then, the infiltration abundance of these cells was tested comparing ccRCC samples and normal control samples, which showed only CD56bright natural killer cell was not statistically significant with a threshold of *p* < 0.05 (Figure 2C and Table S4), meanwhile, univariable Cox analysis indicated that immature dendritic cells (*p*‐value: OS 7.63e−04, PFI 1.71e−04) and central memory CD4 T cells (*p*‐value: OS 0.016, PFI 0.018) were associated with both OS and PFI (Figure 2C).

### 3.2 Immune‐related genes in ccRCC

**FIGURE 3:**
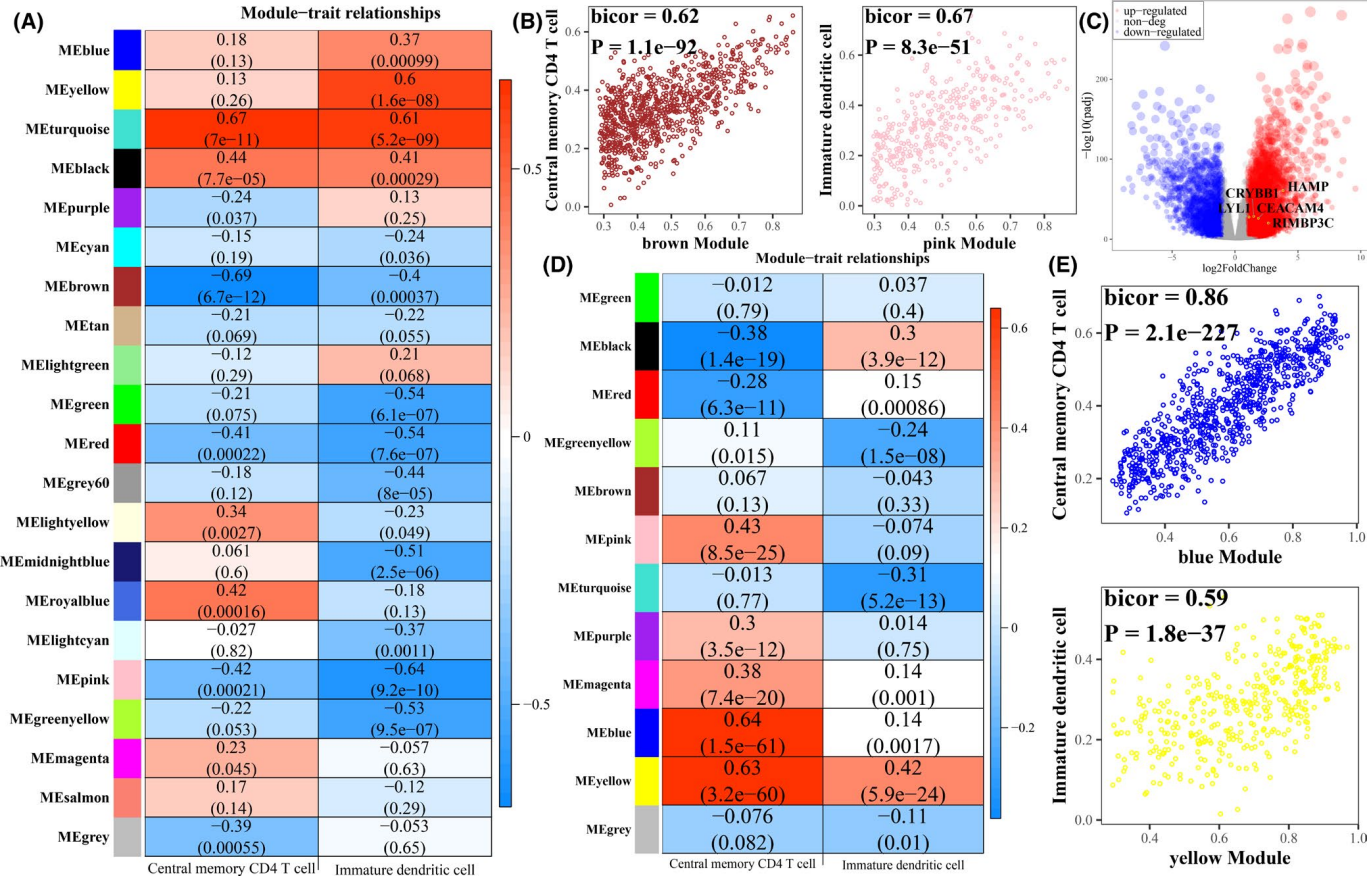
Identification of important immune‐related genes using co‐expression network analysis. (A) The heatmap of the relationships between modules and immune cell infiltrating abundance was investigated in sunitinib‐treated ccRCC, where the color representing the biweight midcorrelation coefficients. Gene numbers in every module (black: 495, blue: 882, brown: 874, cyan: 146, green: 631, green yellow: 224, gray: 5951, gray60: 107, lightcyan: 134, lightgreen: 96, lightyellow: 92, magenta: 251, midnightblue: 143, pink: 378, purple: 226, red: 625, royal blue: 72, salmon: 174, tan: 186, turquoise: 2607, yellow: 706). (B) Gene significance versus module membership. The x‐axis stands for the biweight midcorrelation coefficients between genes expression levels and the corresponding module eigengene, the y‐axis represents the biweight midcorrelation coefficients between genes expression levels with corresponding immune cell abundance. (C) The volcano of differential expression genes between ccRCC and normal control samples with further identified hub genes labeled in yellow circle. (D) The heatmap of the relationships between modules and immune cell infiltrating abundance was investigated in ccRCC, where the color representing the biweight midcorrelation coefficients. Gene numbers in every module (black: 218, blue: 789, brown: 472, green: 304, green yellow: 84, gray: 2543, magenta: 183, pink: 215, purple: 130, red: 237, turquoise: 3187, yellow: 390). (E) Gene significance versus module membership. The x‐axis stands for the biweight midcorrelation coefficients between genes expression levels and the corresponding module eigengene, the y‐axis represents the biweight midcorrelation coefficients between genes expression levels with corresponding immune cell abundance

A co‐expression network including 21 modules (Gene numbers in every module; black: 495, blue: 882, brown: 874, cyan: 146, green: 631, green yellow: 224, gray: 5951, gray60: 107, lightcyan: 134, lightgreen: 96, lightyellow: 92, magenta: 251, midnightblue: 143, pink: 378, purple: 226, red: 625, royal blue: 72, salmon: 174, tan: 186, turquoise: 2607, yellow: 706) was developed in ccRCC samples treated with sunitinib by WGCNA, the clustering dendrogram and topological overlap matrix (TOM) of the network are displayed in Figure S1A–B. Biweight midcorrelation coefficients analysis revealed central memory CD4 T cell was most related to brown module and immature dendritic cell was mainly associated with pink module (Figure 3A). The relationships between MM and GS in both modules were then analyzed, which showed that GS in both immune cells was significantly associated with corresponding MM (Figure 3B). Hence, genes in brown module or pink module were defined as the significant immune‐related genes in sunitinib‐treated ccRCC. On the other hand, a total of 11,200 genes were found to express differentially between ccRCC and normal control samples (Figure 3C), followed by the identification of 12 modules (Gene numbers in every module; black: 218, blue: 789, brown: 472, green: 304, green yellow: 84, gray: 2543, magenta: 183, pink: 215, purple: 130, red: 237, turquoise: 3187, yellow: 390) in the co‐expression network among ccRCC samples from TCGA using WGCNA (Figure S1C,D). As shown in Figure 3D, central memory CD4 T cells and immature dendritic cells were most related to blue and yellow modules respectively, which both indicated a significant correlation between GS and MM (Figure 3E).

### 3.3 Identification of hub genes

**FIGURE 4:**
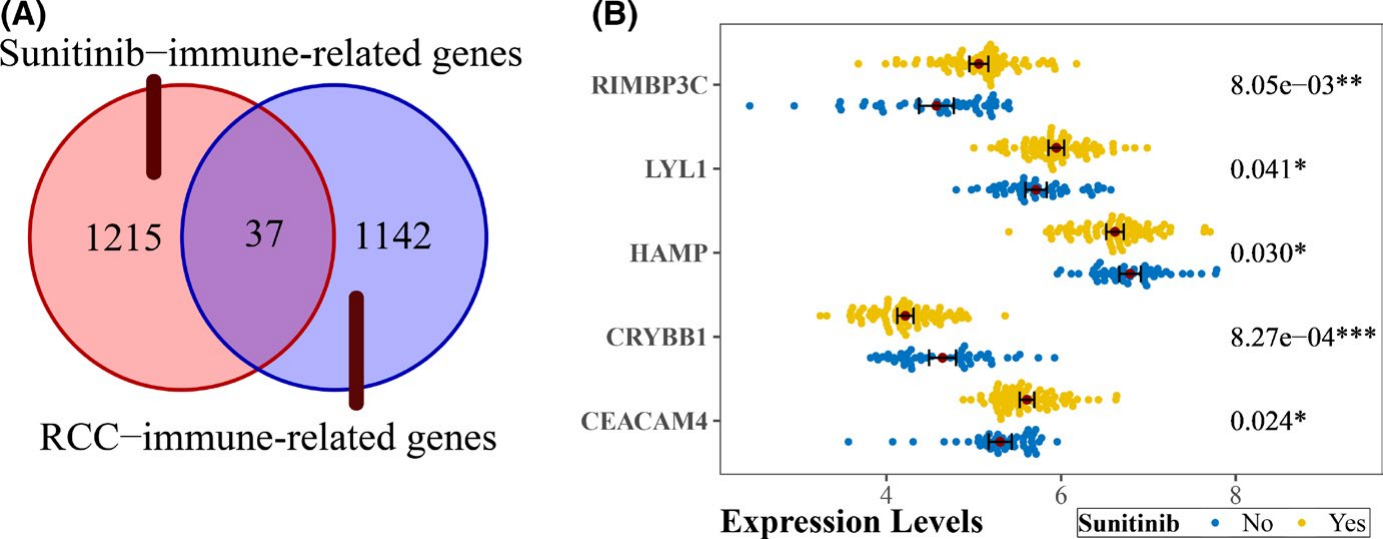
Determination of hub genes related to both sunitinib and immune. (A) Venn diagram for Sunitinib‐immune‐related genes and ccRCC‐immune‐related genes in the co‐expression network of sunitinib‐treated ccRCC from GSE65615 and ccRCC from TCGA, respectively. (B) A beeplot showed differential expression genes between sunitinib‐treated ccRCC with untreated ccRCC, label (*) means *p* < 0.05, label (**) means *p* < 0.01, and label (***) means *p* < 0.001

Immune‐related genes from above both co‐expression networks were first intersected, which generated 37 immune‐related genes (Figure 4A). These genes were then scrutinized for the five differentially expressed genes between sunitinib‐treated ccRCC and untreated ccRCC with a cutoff of adjusted *p* < 0.05 (Figure 4B). These five genes (CRYBB1, RIMBP3C, CEACAM4, HAMP, and LYL1) were defined as hub genes for downstream analysis.

### 3.4 Biological function of hub genes

**FIGURE 5:**
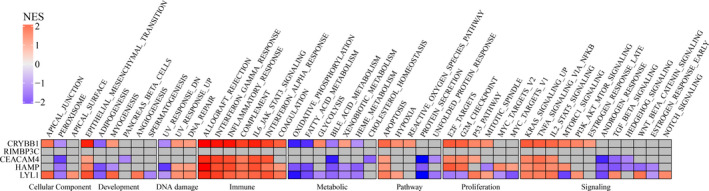
GSEA was applied to explore the associations between hub genes and hallmark gene sets. Normalized enrichment score (NES) is depicted in a heatmap. Gray means statistical insignificance

**FIGURE 6:**
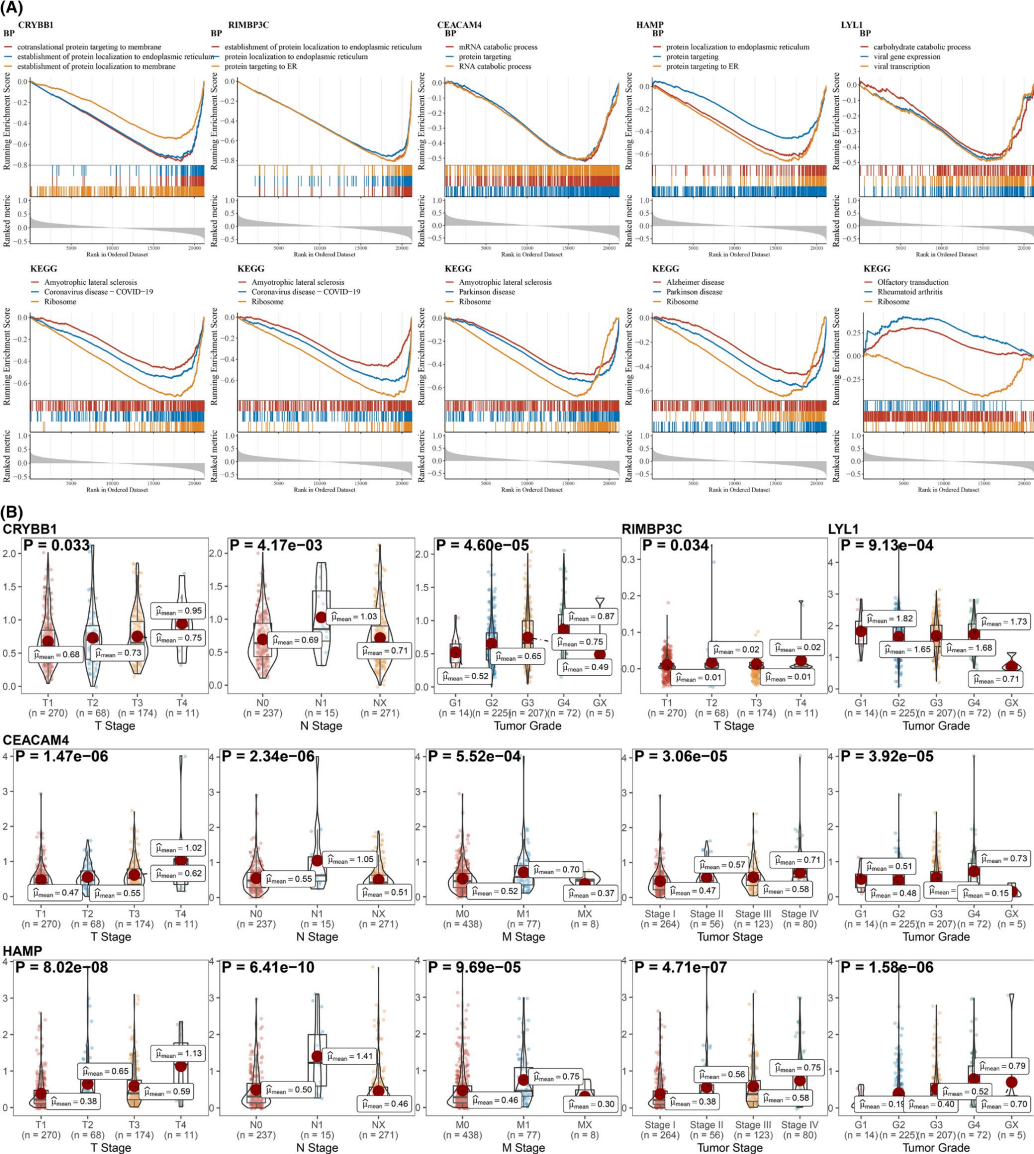
Exploration of the function of hub genes. (A) The top three significant GO analysis results in biological process (BP) and top three significant KEGG analysis results using GSEA are depicted. (B) The correlation between hub genes and tumor stages. The categorical variable with two levels was tested by *t*‐test, and the categorical variable with more than two levels was tested by analysis of variance

GSEA indicated CRYBB1, CEACAM4, HAMP, and LYL1 were highly involved in immune‐, proliferation‐ and signaling‐related pathways (Figure 5). Gene Ontology analysis suggested all hub genes were mostly referred to protein targeting‐related pathways like establishment of protein localization to membrane, protein targeting to ER, and protein targeting process (Figure 6A). KEGG analysis revealed all hub genes were highly related to the ribosome pathway (Figure 6A). The expression levels of hub gene across varied stages of ccRCC were compared, which showed CRYBB1 was significantly correlated with T stage (*p*‐value: 0.033), N stage (*p*‐value: 4.17e−03), and tumor grade (*p*‐value: 4.60e−05), RIMBP3C with T stage (*p*‐value: 0.034), and LYL1 with tumor grade (*p*‐value: 9.13e−04) (Figure 6B and Figure S2). In addition, CEACAM4 and HAMP were revealed to express differently across T stage (*p*‐value; CEACAM4: 1.47e−06; HAMP: 8.02e−08), N stage (*p*‐value; CEACAM4: 2.34e−06; HAMP: 6.41e−10), M stage (*p*‐value; CEACAM4: 5.52e−04; HAMP: 9.69e−05), tumor stage (*p*‐value; CEACAM4: 3.06e−05; HAMP: 4.71e−07), and tumor grade (*p*‐value; CEACAM4: 3.92e−05; HAMP: 1.58e−06) (Figure 6B).

### 3.5 The importance of hub genes to ccRCC

**FIGURE 7:**
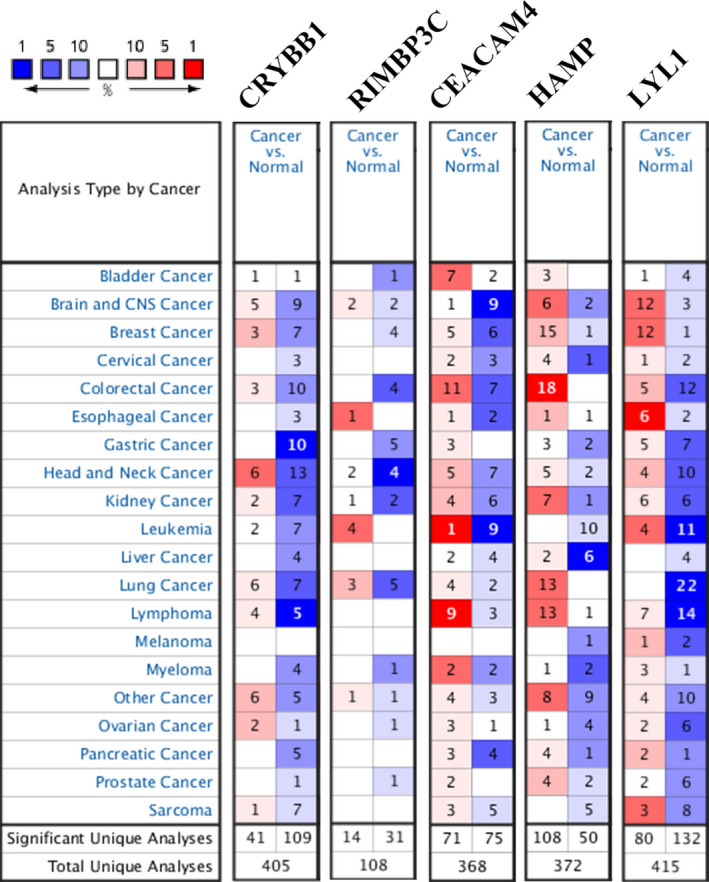
Exploration of expression levels of hub genes across varied types of carcinoma. Red represents over‐expression, and blue stands for down‐expression, the deeper of the color stands for the topper rank of hub genes

**FIGURE 8:**
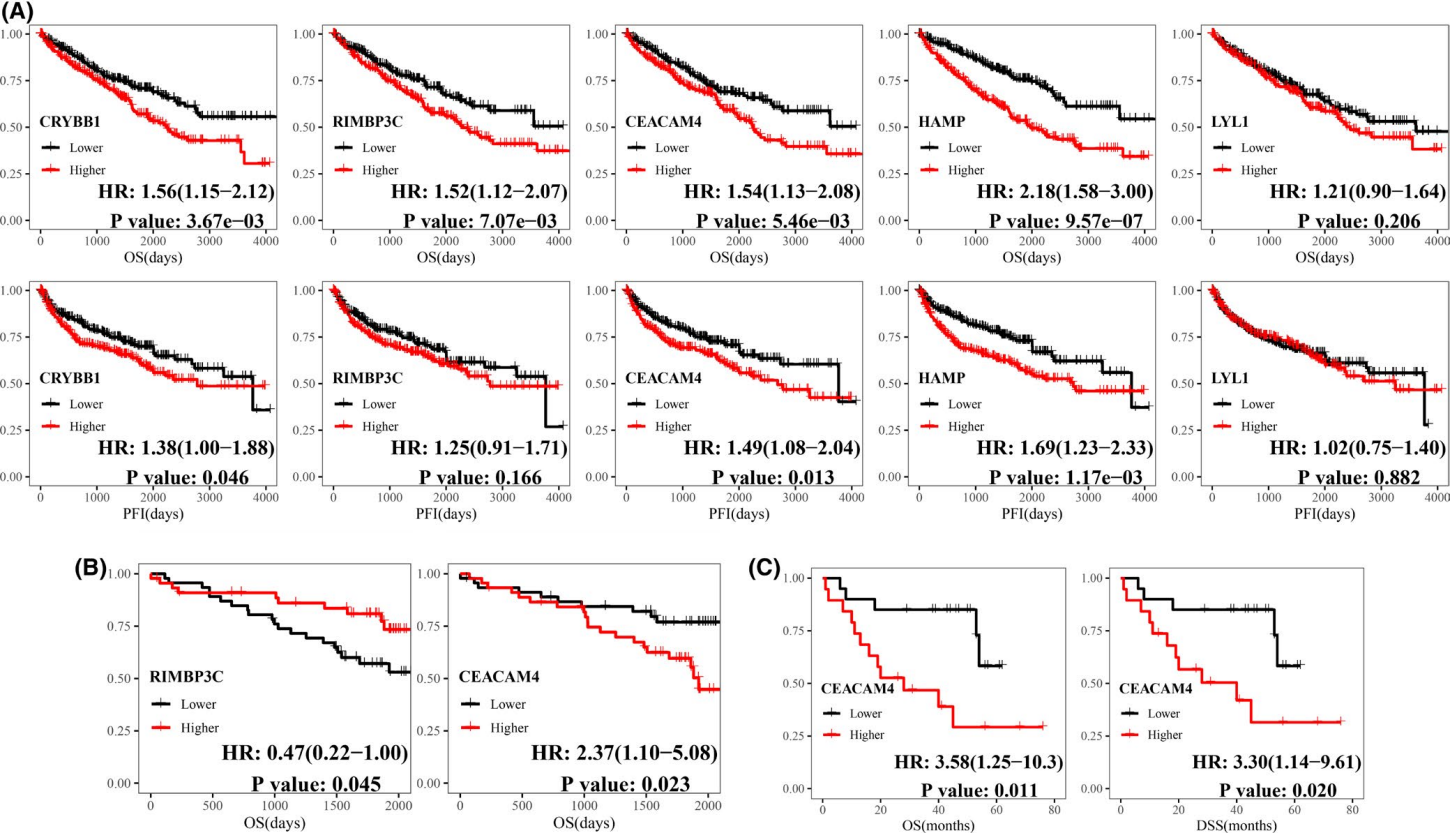
Exploration of the prognostic implications of hub genes. Kaplan–Meier analysis was applied based on the median expression levels of hub genes in (A) TCGA dataset (B) ICGC RECA‐EU dataset, and (C) GSE29609 dataset

Oncomine was searched to verify the significance of all hub genes in each tumor, which presented all hub genes expressed differently across varied tumor sorts when compared with corresponding normal control samples (Figure 7). Log‐rank analysis further indicated CRYBB1 (HR: 1.56 [95% CI: 1.15−2.12]; *p*‐value: 3.67e−03), RIMBP3C (HR: 1.52 [95% CI: 1.12−2.07]; *p*‐value: 7.07e−03), CEACAM4 (HR: 1.54 [95% CI: 1.13−2.08]; *p*‐value: 5.46e−03), and HAMP (HR: 2.18 [95% CI: 1.58−3.00]; *p*‐value: 9.57e−07) were hazard factors to ccRCC with reference to OS in TCGA dataset (Figure 8A) and CRYBB1 (HR: 1.38 [95% CI: 1.00−1.88]; *p*‐value: 0.046), CEACAM4 (HR: 1.49 [95% CI: 1.08−2.04]; *p*‐value: 0.013), and HAMP (HR: 1.69 [95% CI: 1.23−2.33]; *p*‐value: 1.17e−03) were also involved in PFI of ccRCC (Figure 8A). Furthermore, RECA‐EU dataset showed RIMBP3C (HR: 0.47 [95% CI: 0.22−1.00]; *p*‐value: 0.045) and CEACAM4 (HR: 2.37 [95% CI: 1.10−5.08]; *p*‐value: 0.023) were significantly related to OS of ccRCC (Figure 8B and Figure S3A). GSE29609 further gave the same result for CEACAM4 (HR [95% CI]; OS: 3.58 [1.25−10.3], DSS: 3.30 [1.14−9.61]; *p*‐value; OS: 0.011, DSS: 0.020) when referred to OS and DSS (RIMBP3C was not found in GSE29609) (Figure 8C and Figure S3B).

### 3.6 Investigation of sunitinib characteristics of hub genes

**FIGURE 9:**
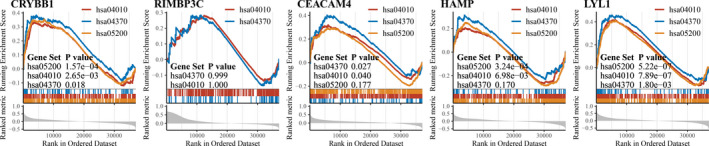
GSEA was used to validate the associations with sunitinib of hub genes. Sunitinib‐related pathways: hsa04370: VEGF signaling pathway; hsa04010: MAPK signaling pathway; and hsa05200: pathways in cancer

Sunitinib‐related pathways (hsa04370: VEGF signaling pathway; hsa04010: MAPK signaling pathway; and hsa05200: pathways in cancer) were analyzed by GSEA, which indicated CRYBB1 (*p*‐value; Pathways in cancer: 1.57e−04; MAPK signaling pathway: 2.65e−03; VEGF signaling pathway: 0.018) and LYL1 (*p*‐value; Pathways in cancer: 5.22e−07; MAPK signaling pathway: 7.89e−07; VEGF signaling pathway: 1.80e−03) were implicated in all sunitinib‐related pathways. In addition, CEACAM4 was enriched in VEGF signaling pathway (*p*‐value: 0.027) and MAPK signaling pathway (*p*‐value: 0.040) and HAMP was significantly associated with pathways in cancer (*p*‐value: 3.24e−04) and MAPK signaling pathway (*p*‐value: 6.98e−03) (Figure 9).

### 3.7 Validation of immune association for both hub genes

**FIGURE 10:**
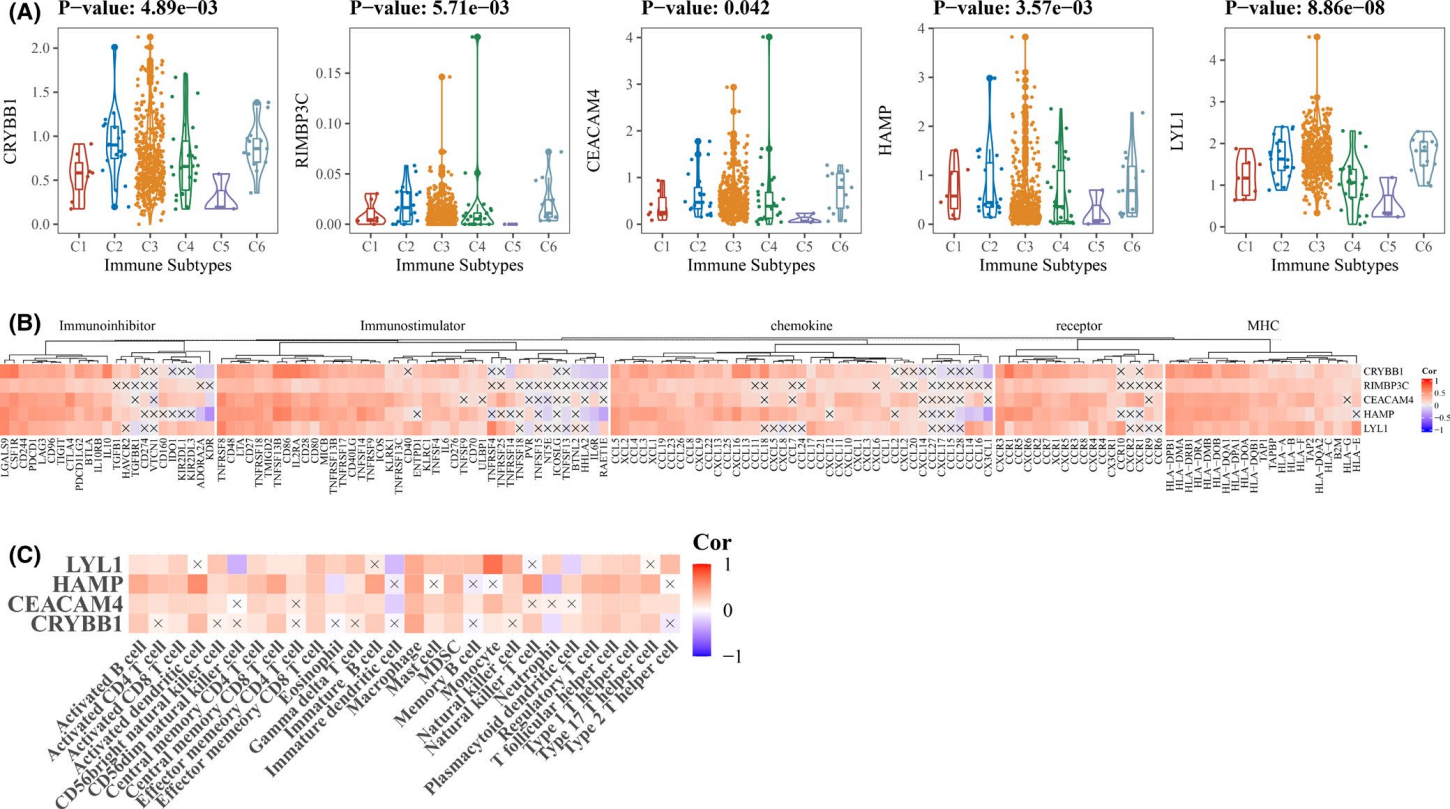
Validation of the associations with immune infiltration. (A) Kruskal–Wallis test was used to analyze the differential expression levels across different immune subtypes. C1 (wound healing); C2 (IFN‐gamma dominant); C3 (inflammatory); C4 (lymphocyte depleted); C5 (immunologically quiet); and C6 (TGF‐b dominant). (B & C) Spearman correlation analysis was utilized to explore the associations between hub genes and (B) immunoinhibitory, immunostimulatory, chemokine, receptor, and MHC or (C) tumor‐infiltrating immune cells

We first confirmed the differential expression of all hub genes across different immune subtypes (Figure 10A) and the correlation with immunoinhibitor, immunomodulators, chemokines, receptor, and MHC (Figure 10B) in TCGA. TISIDB was further employed to authenticate the interaction between hub genes and immune infiltrasion. Spearman correlations analysis indicated that CRYBB1, CEACAM4, HAMP, and LYL1 were positively related to different kinds of tumor‐infiltrating lymphocytes across many tumor types while RIMBP3C showed consistently statistical insignificance (Figure S4). Additionally, we also employed GSE73731 to explore the relationships between hub genes and tumor‐infiltrating lymphocytes which gave similar results for CRYBB1, CEACAM4, HAMP, and LYL1 (RIMBP3C was not found in GSE73731). The abundance of tumor‐infiltrating lymphocytes was estimated by ssGSEA (Table S5). Spearman correlations analysis showed that CRYBB1, CEACAM4, HAMP, and LYL1 were highly positively associated with tumor‐infiltrating lymphocytes in ccRCC (Figure 10C).

## 4 DISCUSSION

RCC constitutes 87% of renal carcinoma which contributed to 73,820 new tumor cases and 14,770 death in the USA in 2019.^47^ Increasing evidences suggested that TME played a vital important role in tumor progression. For another, sunitinib, one of the main tyrosine kinase inhibitors for ccRCC, which has made a huge success in ccRCC treatment, was revealed to act with TME.^5^, ^6^, ^7^ Over here, we identified five sunitinib‐specific hub genes related to TME in ccRCC by ssGSEA, differential expression, and co‐expression network analysis using GSE65615, GTEx, and TCGA data.

Previous studies have shown immune cell as a fundamental ingredient of the TME, and varied immune cells in TME played a vital role in cancer progression and clinical outcomes.^48^ In this study, we refined two types of immune cells (immature dendritic cells and central memory CD4 T cells) which were important to both sunitinib and ccRCC. For the both immune cell types in TME, previous studies showed carcinoma can dislocate the metabolism of dendritic cells and influence their function by nutrient competition and hypoxia, persistent activation of unfolded protein response and lipid uptake.^49^ CD4 +T cells characterized by class II‐restricted and tumor‐specific has been validated to innately infiltrate in TME and exert anticancer duty with the assistance of CD8+ T cells or, with the secretion of type 1 cytokines, or with the direct killing of tumor cells CD4^+^ T cells.^50^ All these studies agree with our findings that immature dendritic cells and central memory CD4 T cells were of vital importance to ccRCC.

Next, immune‐related genes were derived by co‐expression network analysis and differential expression analysis, followed by the identification of five hub genes (CRYBB1, RIMBP3C, CEACAM4, HAMP, and LYL1) related to both sunitinib resistance and immune infiltration in ccRCC. Functional analysis showed that CRYBB1, CEACAM4, HAMP, and LYL1 were implicated in immune‐ and proliferation‐related pathways (Figure 5). Phenotype association analysis suggested all of hub genes involved in tumor development, and it seems CRYBB1, CEACAM4, and HAMP correlated with ccRCC progression more than another two (RIMBP3C and LYL1) (Figure 6B). Survival analysis presented that CRYBB1, RIMBP3C, CEACAM4, and HAMP were highly implicated in the prognosis of ccRCC (Figure 8A). Nevertheless, RECA‐EU and GSE29609 datasets only gave significance to RIMBP3C and CEACAM4, which may be due to small samples in both datasets (Figure 8B,C). GSEA further validated the correlation with sunitinib for CRYBB1, CEACAM4, HAMP, and LYL1 (Figure 9). Tumor microenvironment analysis in TISIDB (Figure S4) and GSE73731 (Figure 10C) confirmed that CRYBB1, CEACAM4, HAMP, and LYL1 were highly related to tumor‐infiltrating lymphocytes. Based on the above perspectives, we inferred that CRYBB1, CEACAM4, and HAMP played a vital role in the tumor progression of ccRCC, the development of sunitinib resistance, and tumor‐infiltrating lymphocytes.

With regard to these five hub genes, there are limited studies associated with CRYBB1 or RIMBP3C. The implication in sunitinib resistance, tumor‐infiltrating, and the progression and development in ccRCC was first uncovered for CRYBB1 and the prognostic implication in ccRCC was also first demonstrated for RIMBP3C in this study. CEACAM4 is a member of carcinoembryonic antigen‐related cell adhesion molecule (CEACAM) family, which is expressed highly in tumors and secreted in serum, and has been widely used as human tumor markers. The CEACAM family is also reported to refer to tumor growth and aggression.^51^, ^52^ Additionally, CEACAM4 which is expressed in primary human granulocytes was reported to be involved in systemic inflammation.^53^, ^54^, ^55^ Moreover, CEACAM4 has been validated to be associated with esophageal squamous cell carcinoma^56^ and medullary thyroid carcinoma.^51^ All of these indicated CEACAM4 was highly related to both immune and tumor. But unfortunately, there were not any laboratory experiments evidence conducted in RCC. HAMP is famous for the maintenance of iron homeostasis^57^, ^58^ and the regulation of cell growth and cycle.^59^ Studies have showed iron metabolism was correlated with inflammation^60^ and malignant tumor, such as multiple myeloma,^61^ hepatocellular carcinoma,^62^ and renal carcinoma.^63^ Moreover, conventional dendritic cells can secrete hepcidin, the product of HAMP, which is noticeable in the inflamed intestine of humans. HAMP has been uncovered to be associated with the prognosis of urothelial carcinoma of the upper urinary tract and RCC^64^ and to contribute to the early stage of carcinogenesis.^59^ HAMP has a strong correlation with immune and carcinoma. Studies concerned about LYL1 mainly focused on lymphoblastic leukemia which indicated LYL1 acted as a oncogene in acute lymphoblastic leukemia and induced the development and progression of acute lymphoblastic leukemia^65^, ^66^, ^67^, ^68^, ^69^ and LYL1 has been demonstrated to regulate the early lymphoid differentiation of immature hematopoietic cells,^70^ which suggested it is possible for the association between immune infiltration and LYL1 in ccRCC.

To the best of our knowledge, our study first explored the potential biomarkers related to immune infiltration and sunitinib resistance for ccRCC based on GEO, GTEx, and TCGA cohorts comprehensively, and further validations were also conducted in KEGG, TISIDB, Oncomine, ICGC, and GEO databases. Nevertheless, there remain some limitations in our study. First, other experimental validations for our findings are in demand and detailed molecular mechanism for the sunitinib‐related and immune infiltrating characteristics in ccRCC has not been investigated; second, other TKIs like axitinib and lenvatinib were not investigated in this study. Therefore, further efforts on the exact molecular mechanism of CRYBB1, RIMBP3C, CEACAM4, HAMP, and LYL1 both in vitro and in vivo are required and further exploration of other TKIs is necessary.

In conclusion, we identified five hub genes (CRYBB1, RIMBP3C, CEACAM4, HAMP, and LYL1) referred to both sunitinib and immune infiltration in ccRCC based on GSE65615, GTEx, and TCGA datasets and further validations refined CRYBB1, CEACAM4, and HAMP which presented a crucial role in the development and progression of ccRCC and implicated in sunitinib resistance and immune infiltrations in ccRCC, which could lead to a better insight into the tumorigenesis and development of ccRCC and the ccRCC‐special TME. Furthermore, CRYBB1, CEACAM4, and HAMP could serve as prognostic biomarkers or potential drug targets for ccRCC, especially for the combination with sunitinib.

## CONFLICT OF INTEREST

None.

## ETHICAL APPROVAL STATEMENT

The approval from the ethics committee and informed consent were waived as the data in this study came from the GEO, TCGA, GTEx, and ICGC databases.

## Supporting information

Fig S1

Fig S2

Fig S3

Fig S4

Table S1

Table S2

Table S3

Table S4

Table S5

## Data Availability

The datasets analyzed for this study can be found in the GTEx (https://www.gtexportal.org/home/datasets), TCGA (https://portal.gdc.cancer.gov/), ICGC (https://dcc.icgc.org/), and GEO (https://www.ncbi.nlm.nih.gov/geo/) databases.
