# Supplementary figures and images for: Key sunitinib‐related biomarkers for renal cell carcinoma
Source: Cancer Med. 2021 Aug 17;10(19):6917–30. doi: 10.1002/cam4.4206 (PMC8495283; doi:10.1002/cam4.4206)

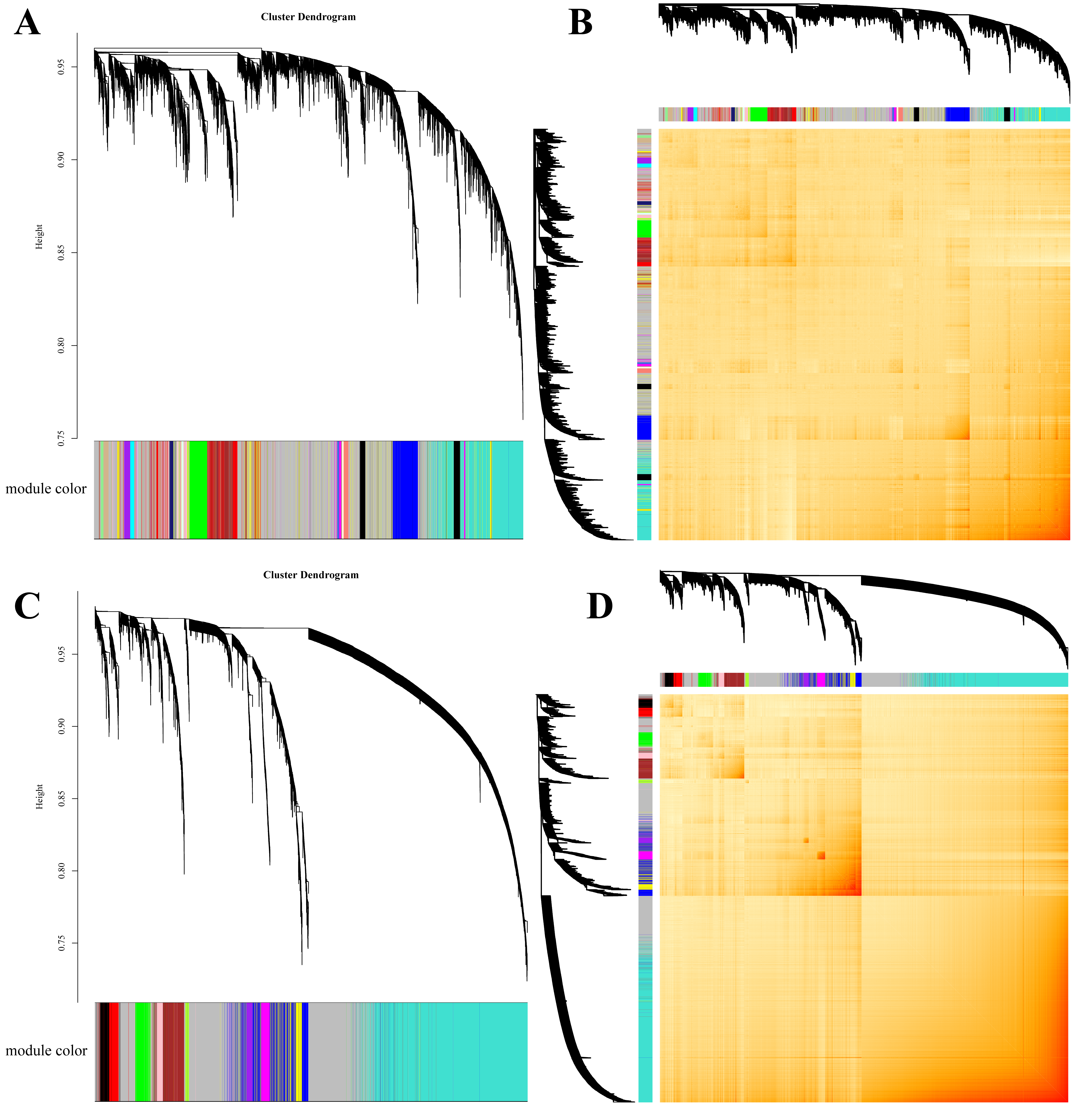

Supplement: Supplementary file 1 — Fig S1 [file CAM4-10-6917-s007.tif]

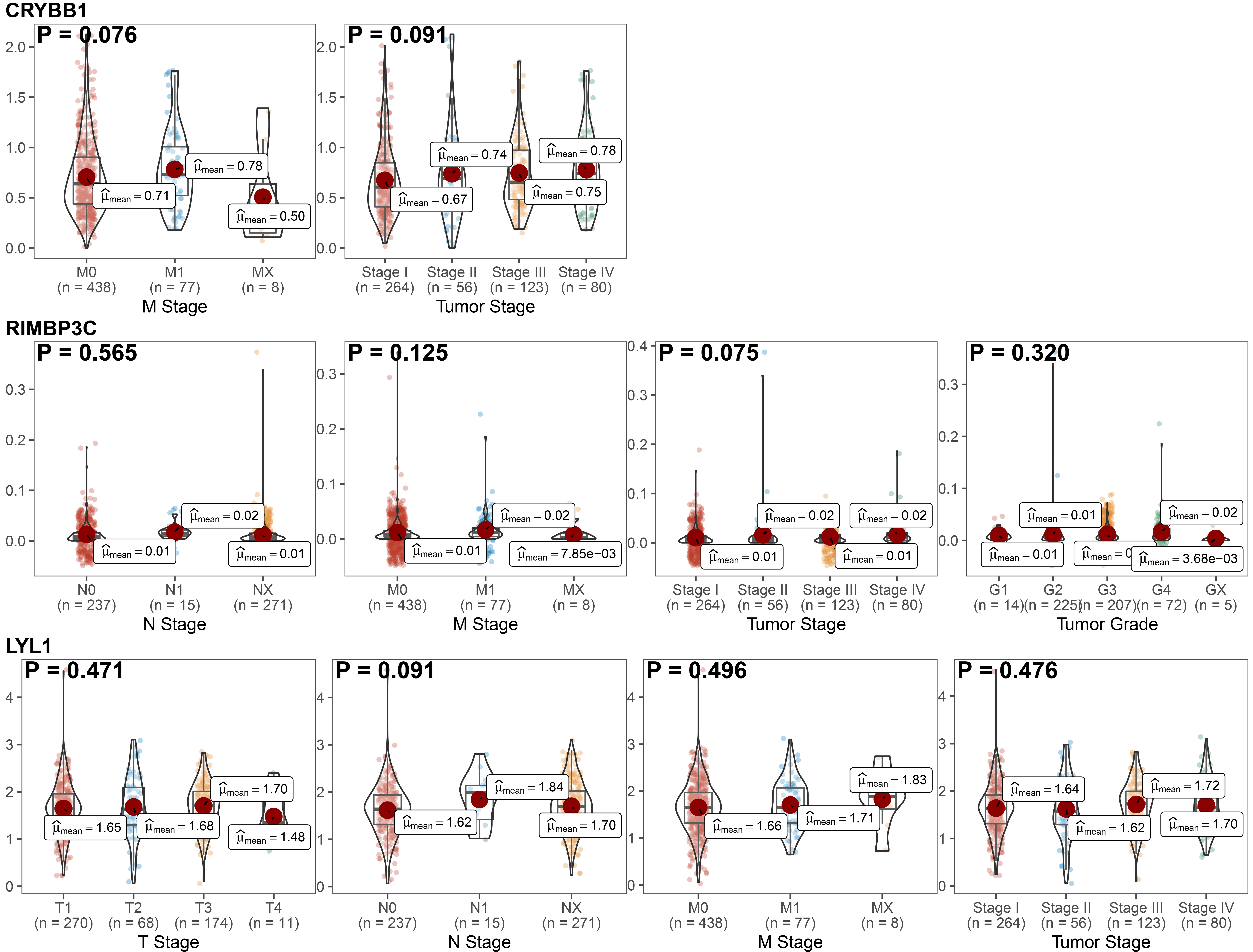

Supplement: Supplementary file 2 — Fig S2 [file CAM4-10-6917-s001.tif]

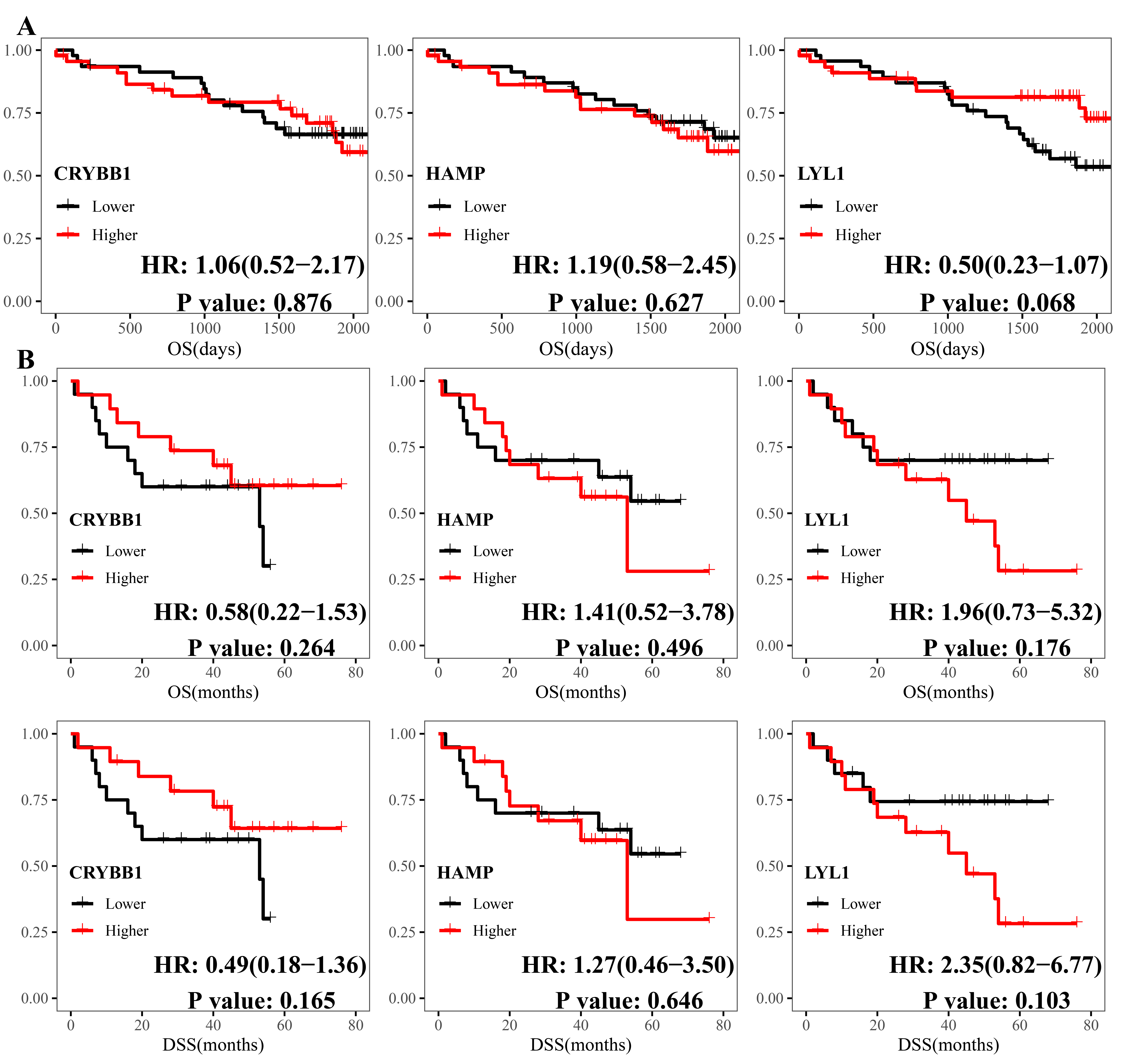

Supplement: Supplementary file 3 — Fig S3 [file CAM4-10-6917-s009.tif]

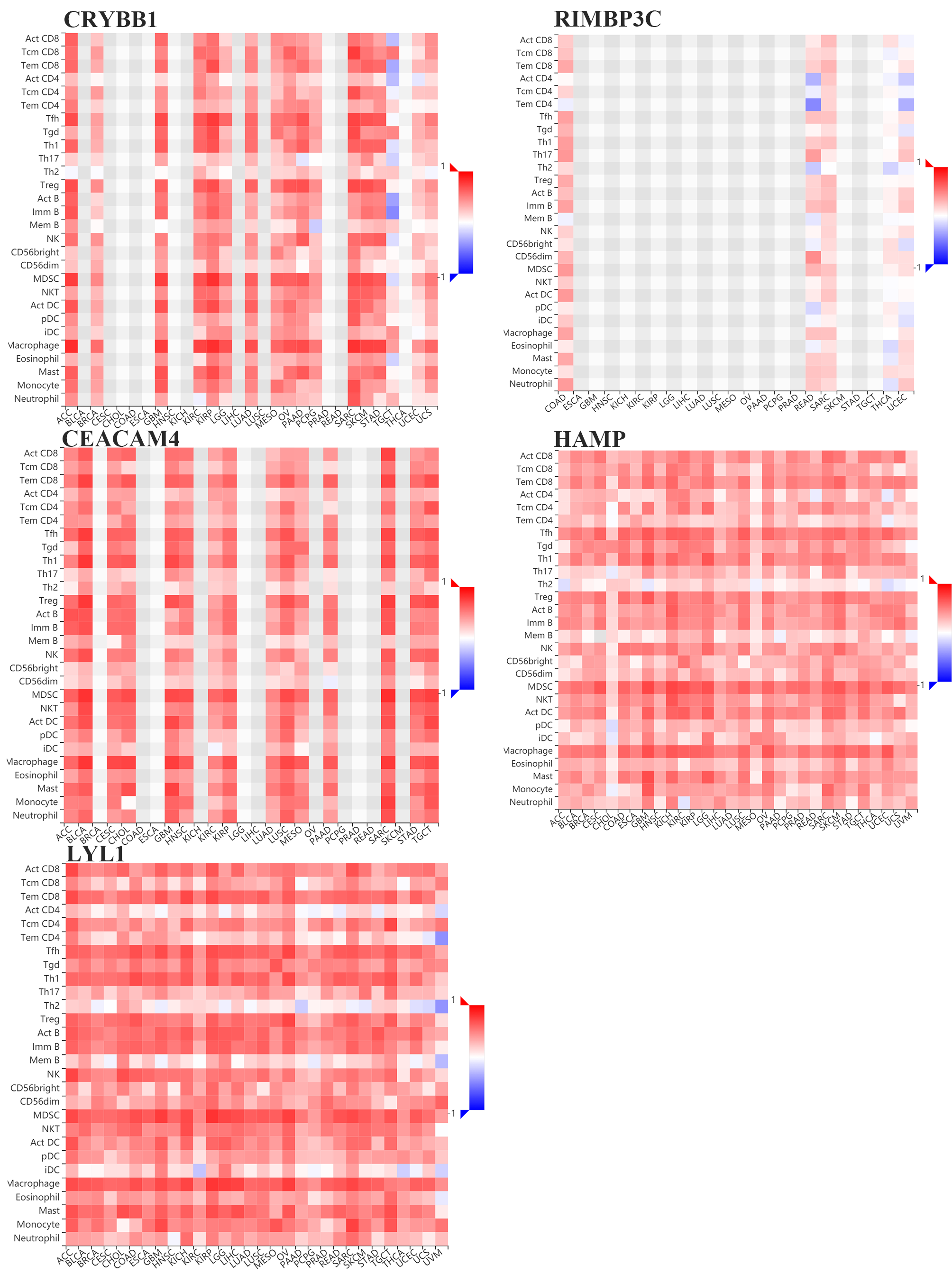

Supplement: Supplementary file 4 — Fig S4 [file CAM4-10-6917-s006.tif]
